# Analysis of the frequency of oncogenic driver mutations and correlation with clinicopathological characteristics in patients with lung adenocarcinoma from Northeastern Switzerland

**DOI:** 10.1186/s13000-019-0789-1

**Published:** 2019-02-11

**Authors:** Alexandra Grosse, Claudia Grosse, Markus Rechsteiner, Alex Soltermann

**Affiliations:** 1 Institute of Pathology and Molecular Pathology, Clinical Pathology, University Hospital Zurich, Rämistrasse 100, 8091 Zurich, Switzerland; 2 Institute of Pathology, Kepler University Hospital, Krankenhausstraße 9, 4021 Linz, Austria; 3 Institute of Pathology and Molecular Pathology, Diagnostic Molecular Pathology, University Hospital Zurich, Rämistrasse 100, 8091 Zurich, Switzerland

**Keywords:** Lung adenocarcinoma, *EGFR*, *KRAS*, *ALK*, *RET*, *ROS1*, *BRAF*, *ERBB2*, *MET*, *PIK3CA*, Non-small cell lung cancer

## Abstract

**Background:**

Molecular testing of lung adenocarcinoma for oncogenic driver mutations has become standard in pathology practice. The aim of the study was to analyze the *EGFR*, *KRAS*, *ALK*, *RET*, *ROS1*, *BRAF*, *ERBB2*, *MET* and *PIK3CA* mutational status in a representative cohort of Swiss patients with lung adenocarcinoma and to correlate the mutational status with clinicopathological patient characteristics.

**Methods:**

All patients who underwent molecular testing of newly diagnosed lung adenocarcinoma during a 4-year period (2014–2018) were included. Molecular analyses were performed with Sanger sequencing (*n* = 158) and next generation sequencing (*n* = 311). *ALK*, *ROS1* and *RET* fusion gene analyses were also performed with fluorescence in situ hybridization and immunohistochemistry/immunocytochemistry. Demographic and clinical data were obtained from the medical records.

**Results:**

Of 469 patients with informative *EGFR* mutation analyses, 90 (19.2%) had *EGFR* mutations. *KRAS* mutations were present in 33.9% of the patients, while 6.0% of patients showed *ALK* rearrangement. *BRAF*, *ERBB2*, *MET* and *PIK3CA* mutations and *ROS1* and *RET* rearrangements were found in 2.6%, 1.9%, 1.9%, 1.5%, 1.7% and 0.8% of the patients, respectively. *EGFR* mutation was significantly associated with female gender and never smoking status. *ALK* translocations were more frequent in never smokers, while *KRAS* mutations were more commonly found in ever smokers. The association between *KRAS* mutational status and female gender was statistically significant only on multivariate analysis after adjusting for smoking.

**Conclusion:**

The *EGFR* mutation rate in the current study is among the higher previously reported mutation rates, while the frequencies of *KRAS*, *BRAF*, *ERBB2* and *PIK3CA* mutations and *ALK*, *ROS1* and *RET* rearrangements are similar to the results of previous reports. *EGFR* and *KRAS* mutations were significantly associated with gender and smoking. *ALK* rearrangements showed a significant association with smoking status alone.

**Electronic supplementary material:**

The online version of this article (10.1186/s13000-019-0789-1) contains supplementary material, which is available to authorized users.

## Background

Lung cancer is the leading cause of cancer-related mortality worldwide [1]. Non-small cell lung cancer (NSCLC) is the most common histological subtype of lung cancer, accounting for approximately 80–85% of lung cancer cases [2, 3]. Molecular testing for epidermal growth factor receptor gene (*EGFR*) mutations and ALK receptor tyrosine kinase (*ALK*) translocations has become the evidence-based standard of care for the management of advanced NSCLC. In the past, pivotal clinical trials have demonstrated clinical benefit from targeting *EGFR* mutations and *ALK* translocations, and currently a number of effective *EGFR* and *ALK* inhibitors are available for targeted therapy of NSCLC harboring the relevant aberrations [4]. More recently, new molecular profiling technologies have permitted the identification of other potential oncogenic drivers including mutations in the KRAS proto-oncogene (*KRAS*), B-Raf proto-oncogene (*BRAF*), erb-b2 receptor tyrosine kinase 2 gene (*ERBB2*), MET proto-oncogene (*MET*) and phosphatidylinositol-3 kinase catalytic subunit alpha gene (*PIK3CA*) as well as ROS proto-oncogene 1 (*ROS1*) and ret proto-oncogene (*RET*) rearrangements [4]. While a number of studies have already evaluated the frequencies of these genetic alterations in NSCLC patients from different countries, information on the prevalence of oncogenic driver mutations in the Swiss population are scarce and limited to population based epidemiological data derived from cancer registries and molecular test results based exclusively on Sanger sequencing rather than next generation sequencing (NGS) [5, 6].

In Switzerland lung cancer is the most common cause of cancer-related death among men (approximately 2000 deaths per year) and the second most common cause of cancer-related death among women (approximately 1100 deaths per year) [7]. Adenocarcinoma is the predominant histological subtype with distinct molecular features, and incidence rates of lung adenocarcinoma are increasing among both sexes [8, 9]. The aim of the study was to analyze the frequencies of *ALK*, *RET* and *ROS1* gene rearrangements and *EGFR*, *KRAS*, *BRAF*, *ERBB2*, *MET* and *PIK3CA* mutations in a representative cohort of Swiss patients with lung adenocarcinoma using NGS as testing method in the majority of cases and to correlate the molecular findings with clinicopathological patient characteristics.

## Methods

### Patients

A total of 475 consecutive patients who underwent molecular testing of newly diagnosed lung adenocarcinoma at the Institute of Pathology and Molecular Pathology, University Hospital Zurich (Zurich, Switzerland), between January 2014 and January 2018, were included in the study, independent of tumor stage. Molecular analyses were performed at the University Hospital Zurich according to National Comprehensive Cancer Network (NCCN) and Swiss Society of Pathology (SSPath) guidelines. Inclusion criteria were histologically and/or cytologically confirmed lung adenocarcinoma, chemotherapy, targeted therapy and radiotherapy naïve, and tissue blocks/cell blocks with adequate tumor cellularity. Exclusion criteria were non-adenocarcinoma histology, previous chemotherapy, targeted therapy or radiotherapy, and insufficient tumor material. Of the initial study population, 469 patients had adequate tumor material for molecular testing, while 6 patients had insufficient tumor samples and were not further evaluated. The results of molecular analysis were recorded for each patient and correlated with demographic and tumor related data such as gender, age, smoking status, clinical stage, and TNM stage (as defined by the Union for International Cancer Control (UICC) TNM classification of malignant tumors, 8th edition [10]). Smoking status was defined as never smokers (< 100 lifetime cigarettes), ex-smokers (≥100 lifetime cigarettes and currently not smoking) and current smokers (≥100 lifetime cigarettes and currently smoking). The cutoff date for data collection was 15 May 2018. The study was approved by the Cantonal Ethics Committee of Zurich (StV-No. 2009/14–0029).

### Molecular analysis

Nucleic acids (DNA and RNA) were isolated from formalin-fixed paraffin-embedded (FFPE) tissue blocks or FFPE cell blocks using the Maxwell 16 FFPE Tissue LEV DNA/RNA Purification Kit (Promega, Fitchburg, WI, USA). The obtained nucleic acids were quantified with NanoDrop ND-1000 spectrophotometer (Thermo Fisher Scientific, Wilmington, DE, USA) and Qubit 2.0 (Thermo Fisher Scientific/Life Technologies, Eugene, OR, USA) using the dsDNA/RNA HS Assay Kit (Thermo Fisher Scientific/Life Technologies, Zug, Switzerland). Mutation analysis was performed using Sanger sequencing (*n* = 158) or NGS (*n* = 311). For DNA- and RNA-based NGS, customer panels including the Ion AmpliSeq Colon and Lung Cancer panel 2 (CLP2), Ion AmpliSeq Fusion Lung Cancer Research panel (LFP), and Oncomine DNA panel for Solid Tumors and Fusion Transcripts (Thermo Fisher Scientific/Life Technologies, Carlsbad, California, USA) were applied, as previously described [11, 12]. Briefly, we used the Ion Library Quantitation kit (Thermo Fisher Scientific) for quantification of DNA and RNA libraries, the Ion One Touch 200 Template Kit v2 DL (lately replaced by the Ion Hi-Q Chef Kit and the Ion Chef System) (Thermo Fisher Scientific) for emulsion polymerase chain reaction (PCR) and template preparation, and the Ion Personal Genome Machine 200 Kit v2 (lately replaced by the Ion Personal Genome Machine Hi-Q Sequencing Kit) (Thermo Fisher Scientific) as the sequencing platform. For Sanger sequencing, we used the Illustra GFX PCR DNA and Gel Band Purification Kit (GE Healthcare Life Sciences, Buckinghamshire, UK) for purification of amplified DNA fragments, the Genetic Analyzer 3130 × 1 (Applied Biosystems, Foster City, CA, USA) for sequencing and the Sequencher 5.1 (Gene Code Corporation, Ann Arbor, MI, USA) for data analysis. *ALK* and *ROS1* immunohistochemistry (IHC)/immunocytochemistry (ICC) was performed on the automated immunostainer DiscoveryUltra (Roche Ventana) using a mouse anti-human *ALK* monoclonal antibody (clone 5A4, Leica Biosystems) and a rabbit anti-human *ROS1* monoclonal antibody (clone D4D6, Cell Signaling Technology). *ALK* or *ROS1* IHC/ICC positive cases were confirmed by fluorescence in situ hybridization (FISH) using the Vysis LSI ALK Dual Color Break Apart Rearrangement Probe (Abbott Molecular, Baar, Switzerland) and the Zyto*Light* SPEC ROS1 Dual Color Break Apart Probe (Zytovision GmbH, Bremerhaven, Germany). FISH testing for *RET* rearrangement was performed using the Zyto*Light* SPEC RET Dual Break Apart Probe (Zytovision GmbH, Bremerhaven, Germany). For each case, a board certified pathologist analyzed 50–100 tumor nuclei. A sample was considered positive, if split signals were detected in ≥15% of tumor nuclei according to the manufacturer’s evaluation guidelines (Abbott Molecular, Des Plaines, IL, USA).

### Statistical analysis

Descriptive statistics were employed to describe the patient characteristics of the study cohort. The results are presented as frequencies and percentages for categorical variables and as mean ± standard deviation, median and range for continuous variables. Associations between mutation status and clinicopathological characteristics were tested using univariate and multivariate analyses. Univariate analysis was performed by chi-square test or Fisher exact test for categorical variables and by t test or nonparametric Mann-Whitney test for continuous variables. Multivariate analysis was performed by logistic regression. *P*-values < 0.05 were considered statistically significant. All statistical analyses were performed using SPSS Statistics software (version 24.0, IBM, Ehningen, Germany).

## Results

The diagnosis of lung adenocarcinoma was based on histology (with or without cytology) in 91.7% (430/469) and on cytology alone in 8.3% (39/469) of the patients. Samples submitted for molecular testing were obtained from primary tumors, lymph node metastases or distant metastases in 79.7% (374/469), 10.7% (50/469) and 9.6% (45/469) of the patients, respectively. There were 191 (40.7%) resection specimens, 224 (47.8%) biopsy specimens, 48 (10.2%) fine needle aspiration/bronchial brushing/bronchoalveolar lavage specimens and 6 (1.3%) cell blocks from pleural effusions. Table 1 summarizes the demographic and clinicopathological patient characteristics. The study population consisted of 235 men and 234 women (mean age at diagnosis, 64.1 ± 11.4 years; range, 27–94 years). The majority of patients were ever smokers (current smokers and ex-smokers) (354/469, 75.5%) and had clinical stage IV lung adenocarcinoma (299/469, 63.8%) at diagnosis. Females were more likely to be never smokers than males (70/234, 29.9% vs 45/235, 19.1%, *p* = 0.007, beta 0.589, OR 1.802, CI 95% 1.174–2.767). Overall 127 patients received targeted treatment. Stage IV patients (both at diagnosis and during follow-up) with *EGFR* mutation and *ALK* rearrangement received targeted treatment in 75.4% and 61.9%, respectively. The majority (91.7%) of stage IV patients with *EGFR* mutation who did not receive targeted therapy were treated with chemotherapy and/or radiotherapy. Likewise, all stage IV patients with *ALK* translocation who were not treated with targeted therapy received chemotherapy and/or radiotherapy. Median patient follow-up was 17 months (range, 1–52 months). 268/469 (57.1%) patients were alive at the time of last follow-up, including 165/469 (35.2%) patients with stable disease and 103/469 (22.0%) patients with progressive disease. 147/469 (31.3%) patients died of disease during follow-up, and 54/469 (11.5%) patients were lost to follow-up. Median overall survival for the entire study cohort was 38 months.

**Table 1:** Patient characteristics

Variable	Study population	Variable	Study population
	(*n* = 469)		(*n* = 469)
Age (years)	64.1 ± 11.4	N stage	
Gender		N0	117 (24.9)
Male	235 (50.1)	N1	65 (13.9)
Female	234 (49.9)	N2	134 (28.6)
Smoking status		N3	153 (32.6)
Never smokers	115 (24.5)	Extrathoracic metastasis/−es	213 (45.4)
Ex-smokers	160 (34.1)	M stage	
Current smokers	194 (41.4)	M0	168 (35.8)
Clinical stage		M1a	88 (18.8)
I	34 (7.2)	M1b	72 (15.4)
II	36 (7.7)	M1c	141 (30.1)
III	100 (21.3)	Localization	
IV	299 (63.8)	Right upper lobe	121 (25.8)
T stage		Right lower lobe	65 (13.9)
T1	68 (14.5)	Middle lobe	19 (4.1)
T1a	9 (1.9)	Left upper lobe	88 (18.8)
T1b	28 (6.0)	Left lower lobe	73 (15.6)
T1c	31 (6.6)	Lingula	14 (3.0)
T2	120 (25.6)	Involvement of two lobes	89 (19.0)
T2a	81 (17.3)	Distribution	
T2b	39 (8.3)	Central	96 (20.5)
T3	93 (19.8)	Peripheral	323 (68.9)
T4	188 (40.1)	Central and peripheral	50 (10.7)
Lymph node metastasis/−es	352 (75.1)	Size (mm)	45 ± 25

Data are mean values ± standard deviations for continuous variables and number of patients with percentages in parentheses for categorical variables

### *EGFR* mutation analysis

A total of 95 *EGFR* mutations were detected in 90/469 (19.2%) patients. The most common *EGFR* mutations were exon 19 deletions (49/90, 54.4%, most frequent subtype: E746_A750del, 33/90, 36.7%) and exon 21 L858R missense mutations (35/90, 38.9%) (Table 2). Doublet *EGFR* mutations were found in 5 (5/90, 5.6%) tumors, including 2 tumors with L858R and non-L858R missense mutations, 2 tumors with two non-L858R missense mutations and 1 tumor with non-L858R missense mutation and T854A primary resistant mutation (Additional file 1: Table S1). The analysis of the distribution of *EGFR* mutations among men and women showed a predominance of *EGFR* mutations in the female group: 55/234 (23.5%) women had a total of 59 *EGFR* mutations (most frequent: exon 19 deletion, 31/55, 56.4%). By contrast, 35/235 (14.9%) males had a total of 36 *EGFR* mutations, the most common of which – as in the female group – were exon 19 deletions (18/35, 51.4%). The highest prevalence of *EGFR* mutations was observed in never smokers (69/115, 60.0%) and was considerably lower in ex-smokers (10/160, 6.3%) and current smokers (11/194, 5.7%). The association between *EGFR* mutational status and either gender or smoking status was statistically significant on univariate (*p* = 0.019 and *p* < 0.001, respectively) and multivariate analyses (*p* = 0.033 and *p* < 0.001, respectively) (Tables 3 and 4). No statistically significant differences were found between *EGFR* mutated and *EGFR* wildtype tumors with respect to clinical stage (except for stage III, *p* = 0.040), T stage (except for T2a, *p* = 0.001), N stage, M stage (except for M1c, *p* = 0.011), tumor location, mean tumor size and mean patient age (Table 3).

**Table 2:** *EGFR* mutations in 90 lung cancers

	cDNA change	Amino acid change	Frequency	Percentage
Exon 21	c.2573 T > G	p.L858R	35	38.9
Exon 19	c.2235_2249del/c.2236_2250del^a^	p.E746_A750del	33	36.7
Exon 19	c.2240_2257del	p.L747_P753delinsS	6	6.7
Exon 19	c.2254_2277del	p.S752_I759del	3	3.3
Exon 19	c.2239_2253del	p.L747_T751del	2	2.2
Exon 19	c.2239_2248delinsC	p.L747_A750delinsP	2	2.2
Exon 19	c.2238_2252delinsGCA	p.L747_T751delinsQ	1	1.1
Exon 19	c.2239_2256del	p.L747_S752del	1	1.1
Exon 19	c.2237_2255delinsT	p.E746_S752delinsV	1	1.1
Exon 18	c.2126A > C	p.E709A	2	2.2
Exon 18	c.2126A > G	p.E709G	1	1.1
Exon 18	c.2156G > C	p.G719A	1	1.1
Exon 18	c.2155G > T	p.G719C	1	1.1
Exon 20	c.2303G > T	p.S768I	2	2.2
Exon 20	c.2320G > A	p.V774 M	1	1.1
Exon 21	c.2497 T > G	p.L833 V	1	1.1
Exon 21	c.2560A > G	p.T854A	1	1.1

^a^c.2235_2249del: *n* = 25; c.2236_2250del: *n* = 8

**Table 3:** Associations between clinicopathological features and *EGFR* mutational status

Variable	*EGFR* wt (*n* = 379)	*EGFR* mt (*n* = 90)	*p*-value
Age (years)	64.1 ± 10.9	64.2 ± 13.1	0.946
Gender			**0.020**
Male	200 (52.8)	35 (38.9)	
Female	179 (47.2)	55 (61.1)	
Smoking status			
Never smokers	46 (12.1)	69 (76.7)	**< 0.001**
Ex-smokers	150 (39.6)	10 (11.1)	**< 0.001**
Current smokers	183 (48.3)	11 (12.2)	**< 0.001**
Clinical stage			
I	25 (6.6)	9 (10.0)	0.263
II	32 (8.4)	4 (4.4)	0.200
III	88 (23.2)	12 (13.3)	**0.040**
IV	234 (61.7)	65 (72.2)	0.063
T stage			
T1	55 (14.5)	13 (14.4)	0.987
T1a	7 (1.8)	2 (2.2)	0.685
T1b	26 (6.9)	2 (2.2)	0.095
T1c	22 (5.8)	9 (10.0)	0.150
T2	90 (23.7)	30 (33.3)	0.061
T2a	55 (14.5)	26 (28.9)	**0.001**
T2b	35 (9.2)	4 (4.4)	0.139
T3	77 (20.3)	16 (17.8)	0.587
T4	157 (41.4)	31 (34.4)	0.224
LN metastasis/−es	289 (76.3)	63 (70.0)	0.218
N stage			
N0	90 (23.7)	27 (30.0)	0.218
N1	57 (15.0)	8 (8.9)	0.129
N2	114 (30.1)	20 (22.2)	0.138
N3	118 (31.1)	35 (38.9)	0.158
Extrathoracic metastasis−/es	164 (43.3)	49 (54.4)	0.056
M stage			
M0	143 (37.7)	25 (27.8)	0.077
M1a	72 (19.0)	16 (17.8)	0.790
M1b	60 (15.8)	12 (13.3)	0.555
M1c	104 (27.4)	37 (41.1)	**0.011**
Localization			
Right upper lobe	95 (25.1)	26 (28.9)	0.456
Right lower lobe	57 (15.0)	8 (8.9)	0.129
Middle lobe	15 (4.0)	4 (4.4)	0.770
Left upper lobe	72 (19.0)	16 (17.8)	0.790
Left lower lobe	60 (18.8)	13 (14.4)	0.744
Lingula	13 (3.4)	1 (1.1)	0.487
Involvement of two lobes	67 (17.7)	22 (24.4)	0.141
Distribution			
Central	74 (19.5)	22 (24.4)	0.298
Peripheral	267 (70.4)	56 (62.2)	0.130
Central and peripheral	38 (10.0)	12 (13.3)	0.361
Size (mm)	45.5 ± 26.3	44.7 ± 20.3	0.744

Data are mean values ± standard deviations for continuous variables and number of patients with percentages in parentheses for categorical variables

Bold numbers indicate significant *p*-values (< 0.05)

**Table 4:** Logistic regression analysis of *EGFR* mutational status

	Univariate logistic regression				Multivariate logistic regression			
	OR	95% CI	Beta	*p*-value	OR	95% CI	Beta	*p*-value
Sex				0.019				0.033
Male	1.00				1.00			
Female	1.756	1.10–2.81	0.563		1.328	0.75–2.37	0.284	
Smoking status				< 0.001				< 0.001
Ever smokers	1.00				1.00			
Never smokers	23.786	13.35–42.38	3.169		23.069	12.92–41.20	3.139	

### *KRAS* mutation analysis

Of 443 patients with informative *KRAS* mutation analysis, 159 (35.9%) harbored *KRAS* mutations. *KRAS* mutations were most frequently located in exon 2 (154/159, 96.9%), and the most common mutations were G12C (72/159, 45.3%) and G12 V (26/159, 16.4%) (Table 5). Of 443 patients with informative *KRAS* and *EGFR* mutation analyses, 2 patients (0.5%) had coexistent *KRAS* and *EGFR* mutations (one with G13S and E746_A750del and one with G12 V and E709A). *KRAS* mutations tended to be more frequent in females (86/217, 39.6%) than in males (73/226, 32.3%) and were more commonly found in ever smokers (152/353, 43.1%) than in never smokers (7/90, 7.8%). The association between *KRAS* mutation and smoking status was statistically significant on both univariate and multivariate analyses after stratification by gender (*p* < 0.001 and *p* < 0.001, beta −2.285, OR 0.102, CI 95% 0.045–0.228), while gender was significantly associated with *KRAS* mutation only on multivariate analysis after adjusting for smoking (*p* = 0.016, beta 0.507, OR 1.660, CI 95% 1.099–2.507) (Tables 6 and 7). Among the patients with informative *KRAS* mutation analysis, males were significantly more likely to be ever smokers (current smokers or ex-smokers) than females (190/226, 84.1% vs 163/217, 75.1%, *p* = 0.020, beta 0.559, OR 1.748, CI 95% 1.092–2.800). No statistically significant differences were found between *KRAS* mutated and *KRAS* wildtype tumors with respect to clinical stage, T stage (except for T2, *p* = 0.043), N stage, M stage, tumor location (except for the left lower lobe, *p* = 0.006), mean tumor size and mean patient age (Table 6).

**Table 5:** *KRAS* mutations in 159 lung cancers

	cDNA change	Amino acid change	Frequency	Percentage
Codon 12/Exon 2	c.34G > T	p.G12C	72	45.3
Codon 12/Exon 2	c.35G > T	p.G12 V	26	16.4
Codon 12/Exon 2	c.35G > A	p.G12D	20	12.6
Codon 12/Exon 2	c.35G > C	p.G12A	15	9.4
Codon 12/Exon 2	c.34_35del	p.G12F	3	1.9
Codon 12/Exon 2	c.34G > C	p.G12R	2	1.3
Codon 12/Exon 2	c.34G > A	p.G12S	1	0.6
Codon 13/Exon 2	c.37G > T	p.G13C	10	6.3
Codon 13/Exon 2	c.37G > A	p.G13S	2	1.3
Codon 13/Exon 2	c.38G > A	p.G13D	2	1.3
Codon 13/Exon 2	c.37G > C	p.G13R	1	0.6
Codon 61/Exon 3	c.183A > C	p.Q61H	3	1.9
Codon 61/Exon 3	c.182A > T	p.Q61L	2	1.3

**Table 6:** Associations between clinicopathological features and *KRAS* mutational status

Variable	*KRAS* wt (*n* = 284)	*KRAS* mt (*n* = 159)	*p*-value
Age (years)	64.7 ± 12.0	63.3 ± 9.4	0.193
Gender			0.108
Male	153 (53.9)	73 (45.9)	
Female	131 (46.1)	86 (54.1)	
Smoking status			
Never smokers	83 (29.2)	7 (4.4)	**< 0.001**
Ex-smokers	99 (34.9)	61 (38.4)	0.461
Current smokers	102 (35.9)	91 (57.2)	**< 0.001**
Clinical stage			
I	19 (6.7)	11 (6.9)	0.927
II	24 (8.5)	10 (6.3)	0.412
III	60 (21.1)	38 (23.9)	0.500
IV	181 (63.7)	100 (62.9)	0.860
T stage			
T1	39 (13.7)	26 (16.4)	0.455
T1a	7 (2.5)	2 (1.3)	0.499
T1b	16 (5.6)	11 (6.9)	0.588
T1c	16 (5.6)	13 (8.2)	0.299
T2	78 (27.5)	30 (18.9)	**0.043**
T2a	51 (18.0)	18 (11.3)	0.065
T2b	27 (9.5)	12 (7.5)	0.485
T3	52 (18.3)	37 (23.3)	0.211
T4	115 (40.5)	66 (41.5)	0.835
LN metastasis/−es	212 (74.6)	121 (76.1)	0.734
N stage			
N0	72 (25.4)	38 (23.9)	0.734
N1	33 (11.6)	27 (17.0)	0.114
N2	82 (28.9)	48 (30.2)	0.771
N3	97 (34.2)	46 (28.9)	0.259
Extrathoracic metastasis/−es	124 (43.7)	74 (46.5)	0.559
M stage			
M0	101 (35.6)	59 (37.1)	0.746
M1a	59 (20.8)	26 (16.4)	0.257
M1b	37 (13.0)	30 (18.9)	0.100
M1c	87 (30.6)	44 (27.7)	0.512
Localization			
Right upper lobe	71 (25.0)	44 (27.7)	0.538
Right lower lobe	45 (15.8)	17 (10.7)	0.134
Middle lobe	11 (3.9)	6 (3.8)	0.958
Left upper lobe	52 (18.3)	31 (19.5)	0.759
Left lower lobe	33 (11.6)	34 (21.4)	**0.006**
Lingula	11 (3.9)	3 (1.9)	0.252
Involvement of two lobes	61 (21.5)	24 (15.1)	0.102
Distribution			
Central	64 (22.5)	27 (17.0)	0.165
Peripheral	189 (66.5)	117 (73.6)	0.124
Central and peripheral	31 (10.9)	15 (9.4)	0.624
Size (mm)	45.8 ± 25.8	45.6 ± 25.8	0.937

Data are mean values ± standard deviations for continuous variables and number of patients with percentages in parentheses for categorical variables

Bold numbers indicate significant *p*-values (< 0.05)

**Table 7:** Logistic regression analysis of *KRAS* mutational status

	Univariate logistic regression				Multivariate logistic regression			
	OR	95% CI	Beta	*p*-value	OR	95% CI	Beta	*p*-value
Sex				0.108				0.016
Male	1.00				1.00			
Female	1.376	0.93–2.03	0.319		1.660	1.10–2.51	0.507	
Smoking status				< 0.001				< 0.001
Ever smokers	1.00				1.00			
Never smokers	0.112	0.05–0.25	−2.194		0.102	0.05–0.23	−2.285	

### *ALK* rearrangement analysis

*ALK* rearrangement was detected by FISH (*n* = 20) or NGS (*n* = 8) in 28/376 (7.4%) tumors, including one with coexistent *KRAS* mutation (G12 V). Of the 8 cases with ALK rearrangement diagnosed by NGS, *EML4* exon 13-*ALK* exon 20 fusion gene variant was found in 4 (50.0%) cases, *EML4* exon 6-*ALK* exon 20 fusion gene variant was detected in 3 (37.5%) cases, and *EML4* exon 18-*ALK* exon 20 fusion gene variant was detected in 1 (12.5%) case. There was no significant difference in the frequency of *ALK* rearrangement between males and females (15/198, 7.6% vs 13/178, 7.3%, univariate analysis, *p* = 0.920, multivariate logistic regression, *p* = 0.669) (Tables 8 and 9). By contrast, *ALK* rearrangement was significantly more common in never smokers than in ever smokers (12/84, 14.3% vs 16/292, 5.5%, *p* = 0.007) (Table 8). The association between *ALK* rearrangement and smoking status remained statistically significant on multivariate analysis after adjusting for gender (*p* = 0.008, beta 1.081, OR 2.948, CI 95% 1.323–6.567) (Table 9). Among the patients tested for *ALK* rearrangement, females were more likely to be never smokers than males (49/178, 27.5% vs 35/198, 17.7%, *p* = 0.023, beta 0.570, OR 1.769, CI 95% 1.082–2.892). No statistically significant differences were found between *ALK*-rearranged and *ALK* wildtype tumors with respect to clinical stage (except for stage III, *p* = 0.038), T stage (except for T1b, *p* = 0.025), N stage (except for N2, *p* = 0.003), M stage (except for M1b, *p* = 0.013), tumor location (except for the right upper lobe, *p* = 0.001, and the middle lobe, *p* = 0.019), mean tumor size and mean patient age (Table 8).

**Table 8:** Associations between clinicopathological features and *ALK* rearrangement

Variable	*ALK* neg. (*n* = 348)	*ALK* pos. (*n* = 28)	*p*-value
Age (years)	64.4 ± 11.2	61.7 ± 14.1	0.229
Gender			0.920
Male	183 (52.6)	15 (53.6)	
Female	165 (47.4)	13 (46.4)	
Smoking status			
Never smokers	72 (20.7)	12 (42.9)	**0.007**
Ex-smokers	124 (35.6)	9 (32.1)	0.710
Current smokers	152 (43.7)	7 (25.0)	0.054
Clinical stage			
I	22 (6.3)	2 (7.1)	0.697
II	29 (8.3)	2 (7.1)	0.822
III	67 (19.3)	10 (35.7)	**0.038**
IV	230 (66.1)	14 (50.0)	0.086
T stage			
T1	47 (13.5)	6 (21.4)	0.258
T1a	8 (2.3)	0 (0.0)	0.263
T1b	19 (5.5)	5 (17.9)	**0.025**
T1c	20 (5.7)	1 (3.6)	0.608
T2	88 (25.3)	7 (25.0)	0.973
T2a	59 (17.0)	4 (14.3)	0.711
T2b	29 (8.3)	3 (10.7)	0.721
T3	70 (20.1)	4 (14.3)	0.455
T4	143 (41.1)	11 (39.3)	0.852
LN metastasis/−es	259 (74.4)	24 (85.7)	0.183
N stage			
N0	89 (25.6)	4 (14.3)	0.183
N1	50 (14.4)	1 (3.6)	0.151
N2	93 (26.7)	15 (53.6)	**0.003**
N3	116 (33.3)	8 (28.6)	0.606
Extrathoracic metastasis/−es	161 (46.3)	7 (25.0)	0.029
M stage			
M0	116 (33.3)	14 (50.0)	0.074
M1a	71 (20.4)	7 (25.0)	0.564
M1b	56 (16.1)	0 (0.0)	**0.013**
M1c	105 (30.2)	7 (25.0)	0.565
Localization			
Right upper lobe	96 (27.6)	0 (0.0)	**0.001**
Right lower lobe	51 (14.7)	5 (17.9)	0.587
Middle lobe	11 (3.2)	4 (14.3)	**0.019**
Left upper lobe	66 (19.0)	6 (21.4)	0.750
Left lower lobe	43 (12.4)	6 (21.4)	0.236
Lingula	11 (3.2)	1 (3.6)	0.611
Involvement of two lobes	70 (20.1)	6 (21.4)	0.868
Distribution			
Central	70 (20.1)	8 (28.6)	0.288
Peripheral	244 (70.1)	16 (57.1)	0.153
Central and peripheral	34 (9.8)	4 (14.3)	0.508
Size (mm)	44.5 ± 24.4	46.0 ± 31.7	0.761

Data are mean values ± standard deviations for continuous variables and number of patients with percentages in parentheses for categorical variables

Bold numbers indicate significant *p*-values (< 0.05)

**Table 9:** Logistic regression analysis of *ALK* rearrangement

	Univariate logistic regression				Multivariate logistic regression			
	OR	95% CI	Beta	*p*-value	OR	95% CI	Beta	*p*-value
Sex				0.920				0.669
Male	1.00				1.00			
Female	0.961	0.44–2.08	−0.040		0.842	0.38–1.85	−0.172	
Smoking status				0.009				0.008
Ever smokers	1.00				1.00			
Never smokers	2.875	1.30–6.35	1.056		2.948	1.32–6.57	1.081	

*KRAS* mutation analysis was not performed in 26 patients with proven *EGFR* mutation, and *ALK* rearrangement testing was not performed in 93 patients including 62 patients with *KRAS* mutation and 31 patients with *EGFR* mutation. Because genetic alterations in *EGFR*, *KRAS* and *ALK* are generally mutually exclusive, it can be concluded that 90/469 (19.2%) patients had *EGFR* mutations, 159/469 (33.9%) had KRAS mutations, and 28/469 (6.0%) had *ALK* gene rearrangement (Table 10). 195/469 (41.6%) patients had triple-negative (*EGFR*-negative/*KRAS*-negative/*ALK*-negative) lung adenocarcinomas.

**Table 10:** Frequency of oncogenic driver mutation in our study cohort

	Study population (n = 469)	Patients tested^a^
*EGFR*	90/469 (19.2)	90/469 (19.2)
*KRAS*	159/469 (33.9)	159/443 (35.9)
*ALK*	28/469 (6.0)	28/376 (7.4)
*BRAF*	12/469 (2.6)	12/309 (3.9)
*ERBB2*	9/469 (1.9)	9/286 (3.1)
*MET*	9/469 (1.9)	9/234 (3.8)
*PIK3CA*	7/469 (1.5)	7/163 (4.3)
*RET*	4/469 (0.8)	4/208 (1.9)
*ROS1*	8/469 (1.7)	8/248 (3.2)

Data are absolute number of patients with percentages in parentheses

^a^Percentages in parentheses refer to the number of tested patients

### *EGFR*, *KRAS* and *ALK* comparative analyses

Comparative analyses of *EGFR* and *KRAS* mutated tumors, *EGFR* mutated and *ALK* rearranged tumors and *KRAS* mutated and *ALK* rearranged tumors are summarized in Additional file 1: Tables S2–S4. Of note, *EGFR* mutated tumors were more likely to have multiple extrathoracic metastases (M1c) compared with *KRAS* mutated tumors (37/90, 41.1% vs 44/159, 27.7%, *p* = 0.030) (Additional file 1: Table S2). *EGFR* mutated tumors were also more likely to be clinical stage IV and have single or multiple extrathoracic metastases (M1b or M1c) compared with *ALK* rearranged tumors (65/90, 72.2% vs 14/28, 50.0%, *p* = 0.029 and 49/90, 54.4% vs 7/28, 25.0%, *p* = 0.006) (Additional file 1: Table S3). *ALK* rearranged tumors were more frequently associated with clinical stage III than *EGFR* mutated tumors (10/28, 35.7% vs 12/90, 13.3%, *p* = 0.008) and more commonly showed ipsilateral mediastinal or subcarinal lymph node metastasis (N2) compared with *EGFR* and *KRAS* mutated lung adenocarcinomas (15/28, 53.6% vs 20/90, 22.2%, *p* = 0.002 and 15/28, 53.6% vs 48/159, 30.2%, *p* = 0.016).

### Other mutations and rearrangements

*RET* fusions were detected by FISH in 4 (1.9%) of 208 tested patients, including 1 patient with coexistent *PIK3CA* mutation (E545K). *ROS1* fusions were detected by FISH (*n* = 5) or NGS (*n* = 3) in 8 (3.2%) of 248 tested patients. No statistically significant differences were found between *RET*/*ROS1* rearranged and non-rearranged tumors with respect to gender, smoking status, clinical stage, TNM stage, tumor location, mean tumor size and mean patient age. 12/309 (3.9%) tumors harbored *BRAF* mutations. The majority of *BRAF* mutations were located in exon 15 (10/12, 83.3%); the most common *BRAF* mutation was V600E (9/12, 75.0%) (Table 11). No statistically significant differences were found between *BRAF* mutated and *BRAF* wildtype tumors with respect to the clinicopathological parameters evaluated. *ERBB2* mutations were detected in 9/286 (3.1%) tumors (Table 12), including 7 insertion/duplication mutations in exon 20, 1 nonsense mutation in exon 13 and 1 missense mutation in exon 8 of the *ERBB2* gene; the most frequent *ERBB2* mutation was p.A775_G776insYVMA (alternative nomenclature p.Y772_A775dup, c.2313_2324dup) (5/9, 55.6%). *ERBB2* mutations were more common in never smokers than in ex−/current smokers (5/64, 7.8% vs 4/222, 1.8%, chi-square test, *p* = 0.029, multivariate logistic regression, *p* = 0.020, beta 1.621, OR 5.059, CI 95% 1.296–19.747), while no significant differences were found between *ERBB2* mutated and *ERBB2* wildtype tumors with respect to the other clinicopathological parameters analyzed. Nine *MET* exon 14 skipping mutations were detected in 234 (3.8%) tumors, including one with coexistent *BRAF* (V600E) and *PIK3CA* (E542K) mutation and one with coexistent *KRAS* (G13C) mutation. *PIK3CA* mutations were detected in 7/163 (4.3%) tumors (3 x E542K, 2 x E545K, 1 x R38H, 1 x H1047R), including one with coexistent *BRAF* (V600E) and *MET* exon 14 skipping mutation, two with coexistent *EGFR* mutations (L858R and L747_P753delinsS, respectively), one with coexistent *KRAS* (G12A) mutation and one with coexistent *RET* rearrangement. No statistically significant differences were found between *MET*/*PIK3CA* mutated and *MET*/*PIK3CA* wildtype tumors with respect to gender, smoking status, clinical stage, TNM stage, mean tumor size and mean patient age. While *MET* mutated tumors were more likely to be located in the right upper lobe than *MET* wildtype tumors (6/9, 66.7% vs 50/225, 22.2%, *p* = 0.007), *PIK3CA* mutated tumors were less likely peripheral in location and involved more frequently both the central and peripheral portions of the lung compared with *PIK3CA* wildtype tumors (2/7, 28.6% vs 107/156, 68.6%, *p* = 0.041 and 3/7, 42.9% vs 10/156, 6.4%, *p* = 0.012). Among the 469 study patients, 154 (32.8%) had lung adenocarcinomas that were negative for all oncogenic driver mutations evaluated in the current study.

**Table 11:** *BRAF* mutations in 12 lung cancers

	cDNA change	Amino acid change	Frequency	Percentage
Exon 15	c.1799 T > A	p.V600E	9	75.0
Exon 15	c.1781A > G	p.D594G	1	8.3
Exon 11	c.1406G > T	p.G469 V	1	8.3
Exon 11	c.1406G > C	p.G469A	1	8.3

**Table 12:** *ERBB2* mutations in 9 lung cancers

ERBB2 mutation type	Mutation	Alternate nomenclature (based on HGVS guidelines)	Frequency	Percentage
Exon 20 insertion	p.A775_G776insYVMA (c.2324_2325ins12)	p.Y772_A775dup (c.2313_2324dup)	5	55.6
Exon 20 insertion	p.P780_Y781insGSP (c.2339_2340insGGCTCCCCA)	p.G778_P780dup (c.2331_2339dup)	1	11.1
Exon 20 insertion	p.G776 > VC (c.2326_2327insTGT)	p.G776delinsVC (c.2326_2327insTGT)	1	11.1
Exon 8 missense mutation	p.Q527* (c.1579C > T)	p.Gln527Ter (c.1579C > T)	1	11.1
Exon 13 nonsense mutation	p.S310Tyr (c.929C > A)	p.Ser310Tyr (c.929C > A)	1	11.1

*HGVS* Human Genome Variation Society

## Discussion

This study presents for the first time data on the *EGFR*, *KRAS*, *ALK*, *ROS1*, *RET*, *BRAF*, *ERBB2*, *MET* and *PIK3CA* mutation frequencies in a representative Swiss cohort of patients with stage I-IV lung adenocarcinoma using NGS as testing method in the majority of patients. Molecular testing was performed in all patients at the time of initial diagnosis during a 4-year period at a primary referral center for lung diseases in Northeastern Switzerland. We also comprehensively studied types of mutations and associations of mutational status with demographic and clinicopathological patient characteristics.

The reported *EGFR* mutation rate in patients with lung adenocarcinoma varies widely between different populations worldwide, ranging from 10 to 20% in European and North American cohorts [5, 6, 13–23] to more than 50% in Asian populations [24, 25]. The wide range of reported *EGFR* mutation rates among European cohorts might be explained by differences between the published studies with respect to patient selection criteria and methods used for molecular analysis. In a French study by Vallee et al. [19], one of the largest single center studies in Europe, *EGFR* mutations were detected in 13.5% of patients with NSCLC and in 14.7% of patients with lung adenocarcinomas. The authors used allele-specific PCR for evaluation of L858R point mutation and DNA fragment analysis to detect exon 19 deletions. Because other *EGFR* mutations were not evaluated, the true prevalence of *EGFR* mutations in this study remains unknown. The INSIGHT study, a large multicenter study comprising 1785 NSCLC patients (including 1393 patients with lung adenocarcinoma), showed an *EGFR* mutation frequency of 13.8% in NSCLC patients and of 15.4% in patients with lung adenocarcinoma [14]. The study analyzed tumor samples from 14 cancer centers in six Central European countries, each with different patient inclusion criteria and testing methods, which makes comparison with other studies more difficult. In addition, mutation testing was not performed at a fixed time point, which could induce bias as mutations may arise during the disease course [23]. Our study results show a prevalence of *EGFR* mutations that is similar to that reported by Moiseyenko et al. [20] (19.8%) in a Russian cohort and by Hlinkova et al. [22] (20%) in a Slovakian cohort, but lower than the *EGFR* mutation rates reported in two previous studies from Switzerland [5, 6]. Ess et al. [5] retrospectively analyzed population based data on the frequency of molecular testing, factors affecting testing and the prevalence of *EGFR* mutations and *ALK* rearrangements in patients with stage IV or relapsed non-squamous NSCLC (including adenocarcinoma, large cell carcinoma and NOS histology) from 2008 to 2014. Using direct sequencing (*EGFR* exons 18–21) for *EGFR* mutation analysis and FISH with a break-apart probe for *ALK* rearrangement testing, *EGFR* mutations (exclusively exon 19 deletions and exon 21 L858R mutations!) were detected in 11% of patients with advanced non-squamous NSCLC and in 13% of patients with lung adenocarcinoma, while 12% of patients with non-squamous NSCLC and 10% of patients with lung adenocarcinoma harbored *ALK* rearrangements. Other oncogenic driver mutations or associations between *EGFR* mutation/*ALK* rearrangement status and clinicopathological characteristics of patients with lung adenocarcinoma were not evaluated. More recently, Schwegler et al. [6] prospectively analyzed population based epidemiological data on overall survival of patients with mutated stage IV lung adenocarcinoma, mostly residents in rural areas of Central Switzerland, from 2010 to 2014. *EGFR* mutations were detected with Sanger sequencing in 14% of the patients, while *KRAS*, *ERBB2*, *BRAF* and *MET* mutations and *ALK* and *RET* translocations were found in 20%, 2%, 1%, 0.5%, 6% and 0.5%, respectively [6]. In contrast to our study, the types of mutations were not analyzed, and mutational status was not correlated with demographic or clinicopathological features. Possible reasons for the reported lower *EGFR* mutation rates compared with that of our study may be different modes of patient selection (selection from the molecular database of University Hospital Zurich vs selection from cancer registries), different patient selection criteria (patients with stage I-IV lung adenocarcinoma vs patients with stage IV or relapsed non-squamous NSCLC [5] and patients with stage IV lung adenocarcinoma [6]) and different methods used for mutational analysis (NGS and Sanger sequencing vs Sanger sequencing alone). In contrast to the studies by Ess et al. [5] and Schwegler et al. [6], the majority of patients in our study underwent molecular testing with NGS, which has been shown to demonstrate high analytic sensitivity, accurate detection of complex indel mutations, and broad reportable ranges with simultaneous detection of doublet *EGFR* mutations and concomitant *KRAS* and *BRAF* mutations in the clinical diagnostic setting [2, 26]. In addition, in the study by Schwegler et al. [6] patients with stage I-III lung adenocarcinoma were excluded from the analysis, and 20% of stage IV lung cancer patients were not tested for oncogenic driver mutations, while in the study by Ess et al. [5] 38% of patients did not receive molecular analysis. Although we did not assess mutation testing rates at our institution, it can be assumed that the molecular testing rates in the period from 2014 to 2018 were higher than those of previous years and that patients treated at an institution active in clinical research are more regularly tested for predictive biomarkers than patients treated at an institution not participating in clinical research [5]. In accordance with published literature [13–16, 23], we found a significant association of *EGFR* mutation status with female gender and never smoking status. When we restricted the analysis to female never smokers, we achieved a high *EGFR* mutation rate of 65.7% (46/70), a finding consistent with previous reports [13, 24, 25].

*KRAS* mutation is one of the most frequent mutations in NSCLC, at least in Caucasian populations, with reported frequencies reaching up to 30% of lung adenocarcinomas [13, 23, 27, 28], while its prevalence in Asian populations is approximately 10% [29–31]. *KRAS* mutations are predominantly found in smokers [32], but they may occur in up to 15% of non-smokers [27]. To date, no effective anti-*KRAS* agent has been released, although a number of preclinical studies and clinical trials are currently underway, exploring novel therapeutic approaches to target *KRAS* mutated NSCLC [33–36]. The *KRAS* mutation rate in our study was slightly higher than was previously reported for Caucasian populations (which might be related to different smoking habits in this Swiss cohort), but was almost identical to the *KRAS* mutation rate reported by Brcic et al. [13] in a Croatian cohort. The presence of *KRAS* mutation in our study was significantly associated with a history of smoking on both univariate and multivariate analyses, while the association of *KRAS* mutation with gender was statistically significant only on multivariate analysis after adjusting for smoking. This finding adds to a mixed body of literature. Some studies have shown increased incidence of *KRAS* mutations among females [23], while others found equal frequencies in both men and women [13, 37, 38].

*ALK* rearrangements are detected in 3–7% of NSCLC [39–44]. They predominantly occur in non-smokers, lung adenocarcinomas and non-Asian vs Asian populations, while men and women seem to be equally affected [3]. The frequency of *ALK* rearrangement in our study is consistent with previous reports, as is the association with smoking status (higher frequency in never smokers). Interestingly, our study showed a higher frequency of ipsilateral mediastinal or subcarinal lymph node metastasis (N2) in *ALK*-rearranged tumors compared with non-rearranged, *EGFR* mutated and *KRAS* mutated tumors, while no significant differences were found between *ALK*-rearranged and non-rearranged/*EGFR* mutated/*KRAS* mutated tumors with regard to N0, N1 and N3 stages. In addition, *ALK* rearranged lung adenocarcinomas were more frequently pT1 tumors compared with *ALK*-non rearranged lung cancer. In a previous study, evaluating surgically resected stage I-III NSCLCs, Paik et al. [45] found that *ALK* FISH-positive NSCLC cases showed lower tumor stage (pT1), but had more frequently lymph node metastases compared with *ALK* FISH-negative NSCLC cases. The authors suggested that *ALK*-rearranged lung cancer might have unique biological features with a tendency to early lymph node metastasis despite the small primary tumor size, which could explain higher incidences of *ALK* rearrangement in advanced NSCLC compared with surgically resectable lung cancer [45].

The frequency of *BRAF* mutations in the current study seems to be among the higher previously reported mutation rates [46–48], but is still lower than the mutation rate reported by Illei et al. [26] (6.3%), who analyzed 1006 lung cancers with NGS. Other targetable genomic alterations in NSCLC, including *RET* and *ROS1* rearrangements and *ERBB2*, *MET* exon 14 skipping and *PIK3CA* mutations, are present only in a small percentage of NSCLC patients (~ 1–2% [49], ~ 2% [50], 2–4% [51–53], 3–4% [54–57] and 2–5% [26, 58, 59], respectively). While our study with a limited sample size of *RET* and *ROS1* rearranged lung cancer showed no significant differences between *RET* or *ROS1* rearranged and non-rearranged tumors regarding clinicopathological characteristics, previous investigations have reported a higher incidence of *RET* and *ROS1* rearrangements in younger age group and never smokers [60, 61] as well as a significant association of *RET* rearranged NSCLC with small primary tumor size and lymph node involvement [60, 62]. According to previous reports [63], *ERBB2* mutations in NSCLC are more common in females, Asian cohorts and never-smokers. While our study showed no significant association of *ERBB2* mutation with female gender, we could confirm the higher prevalence of *ERBB2* mutations in never smoking patients. *PIK3CA* mutations are more commonly encountered in squamous NSCLC [58, 59, 64] and seem to confer inferior prognosis in lung adenocarcinoma [65]. Interestingly, *PIK3CA* mutations have been reported to occur in parallel with other oncogenic driver mutations [66, 67], as was the case in 5 of 7 *PIK3CA* mutated tumors in the present study. Regarding the clinicopathological characteristics of *MET* exon 14 skipping mutation-positive tumors, three retrospective studies showed that *MET* exon 14 skipping positivity in NSCLC patients is significantly associated with advanced age [68–70]. In the current study, we found no significant difference in mean age between patients with and those without *MET* exon 14 mutated tumors. However, we acknowledge that the sample size of *MET* exon 14 mutated tumors was too small to draw meaningful conclusions.

## Conclusion

Our study presents data on the frequency of oncogenic driver mutations in a Northeastern Swiss population with stage I-IV lung adenocarcinoma using NGS as testing method in the majority of cases. A number of studies already analyzed oncogenic driver mutation frequencies, notably *EGFR*, *KRAS* and *ALK* mutation rates, in different populations from European countries. However, based on the available data, the true prevalence of mutations in lung adenocarcinoma is often difficult to determine due to patient selection bias, different testing platforms used for analysis and the histological heterogeneity of tumors included in the studies. Although we cannot exclude some selection toward patients with higher likelihood of mutated tumors in the current study, a major selection bias is unlikely to have occurred because the epidemiological characteristics of our study population are similar to those of the INSIGHT study and other previous investigations. We found a relatively high *EGFR* mutation rate, while *KRAS*, *BRAF*, *ERBB2*, *MET* and *PIK3CA* mutation and *ALK*, *RET* and *ROS1* rearrangement frequencies were similar to those of previous reports. *EGFR* and *KRAS* mutation was significantly associated with gender and smoking status, while *ALK* rearrangement was significantly associated with smoking status alone.

## Additional file

Additional file 1: **Table S1.** Doublet *EGFR* mutations in 90 lung cancers. **Table S2.** Comparison of *EGFR* and *KRAS* mutated tumors. **Table S3.** Comparison of *EGFR* mutated and *ALK* rearranged tumors. **Table S4.** Comparison of *KRAS* mutated and *ALK* rearranged tumors. (DOCX 30 kb)

## Abbreviations

*ALK*: Anaplastic lymphoma kinase
*BRAF*: B rapidly accelerated fibrosarcoma
*EGFR*: Epidermal growth factor receptor
*ERBB2*: Erb-b2 tyrosine kinase 2
FISH: Fluorescence in situ hybridization
HGVS: Human Genome Variation Society
ICC: Immunocytochemistry
IHC: Immunohistochemistry
*KRAS*: Kirsten rat sarcoma
*MET*: Mesenchymal epithelial transition proto-oncogene
NGS: Next generation sequencing
NSCLC: Non-small cell lung cancer
PCR: Polymerase chain reaction
*PIK3CA*: Phosphatidylinositol-3 kinase catalytic subunit alpha
*RET*: Rearranged during transfection proto-oncogene
*ROS1*: ROS proto-oncogene 1
TKI: Tyrosine kinase inhibitor
