# Supplementary material for: Analysis of the frequency of oncogenic driver mutations and correlation with clinicopathological characteristics in patients with lung adenocarcinoma from Northeastern Switzerland
Source: Diagn Pathol. 2019 Feb 11;14:18. doi: 10.1186/s13000-019-0789-1 (PMC6371584; doi:10.1186/s13000-019-0789-1)
Supplement: Supplementary file 1 — Table S1. Doublet EGFR mutations in 90 lung cancers. Table S2. Comparison of EGFR and KRAS mutated tumors. Table S3. Comparison of EGFR mutated and ALK rearranged tumors. Table S4. Comparison of KRAS mutated and ALK rearranged tumors. (DOCX 30 kb) [file 13000_2019_789_MOESM1_ESM.docx]

**Table S1** Doublet *EGFR* mutations in 90 lung cancers

| **Mut 1** | **Exon (mut 1)** | **Mut 2** | **Exon (mut 2)** |
| --- | --- | --- | --- |
| p.L858R | 21 | p.E709G | 18 |
| p.L858R | 21 | p.E709A | 18 |
| p.T854A | 21 | p.V774M | 20 |
| p.G719A | 18 | p.L833V | 21 |
| p.G719C | 18 | p.S768I | 20 |

**Table S2** Comparison of *EGFR* and *KRAS* mutated tumors.

| **Variable** | ***EGFR* mt** (n = 90) | ***KRAS* mt** (n = 159) | ***p*-value** |
| --- | --- | --- | --- |
| Age (years) | 64.2 ± 13.1 | 63.3 ± 9.4 | 0.583 |
| Gender |  |  | 0.283 |
| Male | 35 (38.9) | 73 (45.9) |  |
| Female | 55 (61.1) | 86 (54.1) |  |
| Smoking status |  |  |  |
| Never smokers | 69 (76.7) | 7 (4.4) | **<0.001** |
| Ex-smokers | 10 (11.1) | 61 (38.4) | **<0.001** |
| Current smokers | 11 (12.2) | 91 (57.2) | **<0.001** |
| Clinical stage |  |  |  |
| I | 9 (10.0) | 11 (6.9) | 0.390 |
| II | 4 (4.4) | 10 (6.3) | 0.544 |
| III | 12 (13.3) | 38 (23.9) | **0.046** |
| IV | 65 (72.2) | 100 (62.9) | 0.135 |
| T stage |  |  |  |
| T1 | 13 (14.4) | 26 (16.4) | 0.691 |
| T1a | 2 (2.2) | 2 (1.3) | 0.622 |
| T1b | 2 (2.2) | 11 (6.9) | 0.143 |
| T1c | 9 (10.0) | 13 (8.2) | 0.626 |
| T2 | 30 (33.3) | 30 (18.9) | **0.010** |
| T2a | 26 (28.9) | 18 (11.3) | **<0.001** |
| T2b | 4 (4.4) | 12 (7.5) | 0.337 |
| T3 | 16 (17.8) | 37 (23.3) | 0.309 |
| T4 | 31 (34.4) | 66 (41.5) | 0.272 |
| LN metastasis/-es | 63 (70.0) | 121 (76.1) | 0.292 |
| N stage |  |  |  |
| N0 | 27 (30.0) | 38 (23.9) | 0.292 |
| N1 | 8 (8.9) | 27 (17.0) | 0.078 |
| N2 | 20 (22.2) | 48 (30.4) | 0.175 |
| N3 | 35 (38.9) | 46 (28.9) | 0.107 |
| Extrathoracic metastasis/-es | 49 (54.4) | 74 (46.5) | 0.231 |
| M stage |  |  |  |
| M0 | 25 (27.8) | 59 (37.1) | 0.135 |
| M1a | 16 (17.8) | 26 (16.4) | 0.773 |
| M1b | 12 (13.3) | 30 (18.9) | 0.263 |
| M1c | 37 (41.1) | 44 (27.7) | **0.030** |
| Localization |  |  |  |
| Right upper lobe | 26 (28.9) | 44 (27.7) | 0.838 |
| Right lower lobe | 8 (8.9) | 17 (10.7) | 0.649 |
| Middle lobe | 4 (4.4) | 6 (3.8) | 0.751 |
| Left upper lobe | 16 (17.8) | 31 (19.5) | 0.739 |
| Left lower lobe | 13 (14.4) | 34 (21.4) | 0.179 |
| Lingula | 1 (1.1) | 3 (1.9) | 0.630 |
| Involvement of two lobes | 22 (24.4) | 24 (15.1) | 0.068 |
| Distribution |  |  |  |
| Central | 22 (24.4) | 27 (17.0) | 0.155 |
| Peripheral | 56 (62.2) | 117 (73.6) | 0.061 |
| Central and peripheral | 12 (13.3) | 15 (9.4) | 0.342 |
| Size (mm) | 44.7 ± 20.3 | 45.6 ± 25.8 | 0.756 |

Data are mean values ± standard deviations for continuous variables and number of patients with percentages in parentheses for categorical variables.

Bold numbers indicate significant p-values (< 0.05).

**Table S3** Comparison of *EGFR* mutated and *ALK* rearranged tumors.

| **Variable** | ***EGFR* mt** (n = 90) | ***ALK* pos.** (n = 28) | ***p*-value** |
| --- | --- | --- | --- |
| Age (years) | 64.2 ± 13.1 | 61.7 ± 14.1 | 0.394 |
| Gender |  |  | 0.170 |
| Male | 35 (38.9) | 15 (53.6) |  |
| Female | 55 (61.1) | 13 (46.4) |  |
| Smoking status |  |  |  |
| Never smokers | 69 (76.7) | 12 (42.9) | **0.001** |
| Ex-smokers | 10 (11.1) | 9 (32.1) | **0.016** |
| Current smokers | 11 (12.2) | 7 (25.0) | 0.131 |
| Clinical stage |  |  |  |
| I | 9 (10.0) | 2 (7.1) | 0.641 |
| II | 4 (4.4) | 2 (7.1) | 0.627 |
| III | 12 (13.3) | 10 (35.7) | **0.008** |
| IV | 65 (72.2) | 14 (50.0) | **0.029** |
| T stage |  |  |  |
| T1 | 13 (14.4) | 6 (21.4) | 0.387 |
| T1a | 2 (2.2) | 0 (0.0) | 0.296 |
| T1b | 2 (2.2) | 5 (17.9) | **0.008** |
| T1c | 9 (10.0) | 1 (3.6) | 0.448 |
| T2 | 30 (33.3) | 7 (25.0) | 0.406 |
| T2a | 26 (28.9) | 4 (14.3) | 0.121 |
| T2b | 4 (4.4) | 3 (10.7) | 0.354 |
| T3 | 16 (17.8) | 4 (14.3) | 0.780 |
| T4 | 31 (34.4) | 11 (39.3) | 0.640 |
| LN metastasis/-es | 63 (70.0) | 24 (85.7) | 0.099 |
| N stage |  |  |  |
| N0 | 27 (30.0) | 4 (14.3) | 0.099 |
| N1 | 8 (8.9) | 1 (3.6) | 0.684 |
| N2 | 20 (22.2) | 15 (53.6) | **0.002** |
| N3 | 35 (38.9) | 8 (28.6) | 0.322 |
| Extrathoracic metastasis/-es | 49 (54.4) | 7 (25.0) | **0.006** |
| M stage |  |  |  |
| M0 | 25 (27.8) | 14 (50.0) | 0.029 |
| M1a | 16 (17.8) | 7 (25.0) | 0.399 |
| M1b | 12 (13.3) | 0 (0.0) | 0.067 |
| M1c | 37 (41.1) | 7 (25.0) | 0.124 |
| Localization |  |  |  |
| Right upper lobe | 26 (28.9) | 0 (0.0) | **0.001** |
| Right lower lobe | 8 (8.9) | 5 (17.9) | 0.186 |
| Middle lobe | 4 (4.4) | 4 (14.3) | 0.090 |
| Left upper lobe | 16 (17.8) | 6 (21.4) | 0.665 |
| Left lower lobe | 13 (14.4) | 6 (21.4) | 0.387 |
| Lingula | 1 (1.1) | 1 (3.6) | 0.420 |
| Involvement of two lobes | 22 (24.4) | 6 (21.4) | 0.743 |
| Distribution |  |  |  |
| Central | 22 (24.4) | 8 (28.6) | 0.661 |
| Peripheral | 56 (62.2) | 16 (57.1) | 0.630 |
| Central and peripheral | 12 (13.3) | 4 (14.3) | 0.898 |
| Size (mm) | 44.7 ± 20.3 | 46.0 ± 31.7 | 0.791 |

Data are mean values ± standard deviations for continuous variables and number of patients with percentages in parentheses for categorical variables.

Bold numbers indicate significant p-values (< 0.05).

**Table S4** Comparison of *KRAS* mutated and *ALK* rearranged tumors.

| **Variable** | ***KRAS* mt** (n = 159) | ***ALK* pos.** (n = 28) | ***p*-value** |
| --- | --- | --- | --- |
| Age (years) | 63.3 ± 9.4 | 61.7 ± 14.1 | 0.565 |
| Gender |  |  | 0.454 |
| Male | 73 (45.9) | 15 (53.6) |  |
| Female | 86 (54.1) | 13 (46.4) |  |
| Smoking status |  |  |  |
| Never smokers | 7 (4.4) | 12 (42.9) | **<0.001** |
| Ex-smokers | 61 (38.4) | 9 (32.1) | 0.530 |
| Current smokers | 91 (57.2) | 7 (25.0) | **0.002** |
| Clinical stage |  |  |  |
| I | 11 (6.9) | 2 (7.1) | 0.966 |
| II | 10 (6.3) | 2 (7.1) | 0.696 |
| III | 38 (23.9) | 10 (35.7) | 0.187 |
| IV | 100 (62.9) | 14 (50.0) | 0.197 |
| T stage |  |  |  |
| T1 | 26 (16.4) | 6 (21.4) | 0.586 |
| T1a | 2 (1.3) | 0 (0.0) | 0.419 |
| T1b | 11 (6.9) | 5 (17.9) | 0.070 |
| T1c | 13 (8.2) | 1 (3.6) | 0.698 |
| T2 | 30 (18.9) | 7 (25.0) | 0.453 |
| T2a | 18 (11.3) | 4 (14.3) | 0.749 |
| T2b | 12 (7.5) | 3 (10.7) | 0.475 |
| T3 | 37 (23.3) | 4 (14.3) | 0.289 |
| T4 | 66 (41.5) | 11 (39.3) | 0.826 |
| LN metastasis/-es | 121 (76.1) | 24 (85.7) | 0.261 |
| N stage |  |  |  |
| N0 | 38 (23.9) | 4 (14.3) | 0.261 |
| N1 | 27 (17.0) | 1 (3.6) | 0.084 |
| N2 | 48 (30.2) | 15 (53.6) | **0.016** |
| N3 | 46 (28.9) | 8 (28.6) | 0.969 |
| Extrathoracic metastasis/-es | 74 (46.5) | 7 (25.0) | **0.034** |
| M stage |  |  |  |
| M0 | 59 (37.1) | 14 (50.0) | 0.197 |
| M1a | 26 (16.4) | 7 (25.0) | 0.285 |
| M1b | 30 (18.9) | 0 (0.0) | **0.009** |
| M1c | 44 (27.7) | 7 (25.0) | 0.770 |
| Localization |  |  |  |
| Right upper lobe | 44 (27.7) | 0 (0.0) | **0.001** |
| Right lower lobe | 17 (10.7) | 5 (17.9) | 0.336 |
| Middle lobe | 6 (3.8) | 4 (14.3) | **0.045** |
| Left upper lobe | 31 (19.5) | 6 (21.4) | 0.813 |
| Left lower lobe | 34 (21.4) | 6 (21.4) | 0.996 |
| Lingula | 3 (1.9) | 1 (3.6) | 0.480 |
| Involvement of two lobes | 24 (15.1) | 6 (21.4) | 0.406 |
| Distribution |  |  |  |
| Central | 27 (17.0) | 8 (28.6) | 0.147 |
| Peripheral | 117 (73.6) | 16 (57.1) | 0.077 |
| Central and peripheral | 15 (9.4) | 4 (14.3) | 0.494 |
| Size (mm) | 45.6 ± 25.8 | 46.0 ± 31.7 | 0.938 |

Data are mean values ± standard deviations for continuous variables and number of patients with percentages in parentheses for categorical variables.

Bold numbers indicate significant p-values (< 0.05).
